# AXIN2 promotes degradation of AXIN1 through tankyrase in colorectal cancer cells

**DOI:** 10.1111/febs.17226

**Published:** 2024-07-18

**Authors:** Olivia Schmidt, Martina Brückner, Dominic B. Bernkopf

**Affiliations:** ^1^ Experimental Medicine II, Nikolaus‐Fiebiger‐Center Friedrich‐Alexander University Erlangen‐Nürnberg Germany

**Keywords:** AXIN1, AXIN2, colorectal cancer, TNKS, Wnt pathway

## Abstract

AXIN1 and AXIN2 are homologous proteins that inhibit the Wnt/β‐catenin signaling pathway, which is frequently hyperactive in colorectal cancer. Stabilization of AXIN1 and AXIN2 by inhibiting their degradation through tankyrase (TNKS) allows the attenuation of Wnt signaling in cancer, attracting interest for potential targeted therapy. Here, we found that knockout or knockdown of AXIN2 in colorectal cancer cells increased the protein stability of AXIN1. The increase in AXIN1 overcompensated for the loss of AXIN2 with respect to protein levels; however, functionally it did not because loss of AXIN2 activated the pathway. Moreover, AXIN2 was highly essential in the context of TNKS inhibition because TNKS‐targeting small‐molecule inhibitors completely failed to inhibit Wnt signaling and to stabilize AXIN1 in AXIN2 knockout cells. The increased AXIN1 protein stability and the impaired stabilization by TNKS inhibitors indicated disrupted TNKS‐AXIN1 regulation in AXIN2 knockout cells. Concordantly, mechanistic studies revealed that co‐expression of AXIN2 recruited TNKS to AXIN1 and stimulated TNKS‐mediated degradation of transiently expressed AXIN1 wild‐type and AXIN1 mutants with impaired TNKS binding. Taken together, our data suggest that AXIN2 promotes degradation of AXIN1 through TNKS in colorectal cancer cells by directly linking the two proteins, and these findings may be relevant for TNKS inhibition‐based colorectal cancer therapies.

## Graphical abstract

AXIN1 and AXIN2 are highly related proteins that inhibit the proto‐oncogenic Wnt/β‐catenin pathway. This signaling pathway is frequently activated in colorectal cancer. Both AXIN1 and AXIN2 are degraded through a tankyrase (TNKS)‐dependent mechanism. Here, we show that AXIN2 promotes TNKS‐mediated degradation of AXIN1. Mechanistically, AXIN2 recruits TNKS into AXIN1 condensates, as it interacts with both components. Concordantly, loss of AXIN2 disrupted regulation of AXIN1 expression by TNKS in SW480 colorectal cancer cells, which may have therapeutic implications for colorectal cancer through TNKS inhibitors.

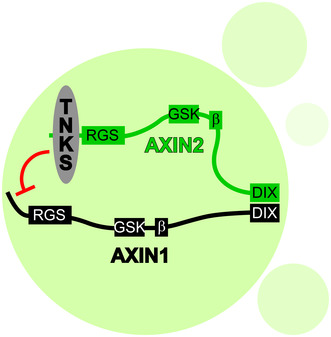

**Abbreviations**
APC: adenomatous polyposis coli
Cas9: CRISPR‐associated 9
CHX: cycloheximide
CRISPR: clustered regularly interspaced short palindromic repeats
DIX: dishevelled and AXIN
FOP: far from optimal
GFP: green fluorescent protein
GSK3B: glycogen synthase kinase 3 beta
HRP: horseradish peroxidase
IF: immunofluorescence
PARsylation: poly‐ADP‐ribosylation
siRNA: small interfering RNA
TNKS: tankyrase
TOP: T cell factor optimal
WB: western blotting
WT: wild‐type

## Introduction

The Wnt/β‐catenin signaling pathway posttranslationally controls the protein levels of the proto‐oncogenic transcriptional co‐factor β‐catenin. In the absence of Wnt ligands, the scaffold proteins AXIN1 and AXIN2 recruit the tumor suppressor adenomatous polyposis coli (APC), casein kinase 1 alpha and glycogen synthase kinase 3 beta (GSK3B), assembling a multiprotein β‐catenin destruction complex [1]. The β‐catenin destruction complex phosphorylates β‐catenin, promoting its consequent ubiquitination and proteasomal degradation. Thus, the transcription of β‐catenin target genes is silenced. Binding of Wnt ligands to frizzled receptors and low‐density lipoprotein receptor‐related protein 5 and 6 co‐receptors inhibits the β‐catenin destruction complex, leading to stabilization and nuclear translocation of β‐catenin [2]. In the nucleus, β‐catenin interacts with transcription factors of the T cell factor/lymphoid enhancer factor family to induce transcription of pro‐proliferative target genes such as *MYC* and *CCND1* [3]. In colorectal cancer, the Wnt signaling transduction cascade is frequently impaired, most commonly through inactivating mutations of the tumor suppressor APC [4], causing insufficient degradation of β‐catenin and pathologically hyperactive transcription of β‐catenin target genes.

AXIN1 and AXIN2 are two homologs proteins sharing a highly conserved domain structure [5, 6]. The two homologs function very similarly as scaffold proteins mediating the assembly of the β‐catenin destruction complex, and thus inhibiting Wnt/β‐catenin signaling [5, 6, 7]. Studies with overexpressed proteins have suggested that AXIN1‐mediated degradation of β‐catenin depends on polymerization of AXIN1 through its C‐terminal dishevelled and AXIN (DIX) domain, giving rise to microscopically visible spherical protein assemblies [8, 9]. Although AXIN2 does usually not form these spheres, it contains a functional DIX domain mediating DIX–DIX polymerization [5, 10]. The most prominent difference between AXIN1 and AXIN2 is their transcriptional regulation. Although *AXIN1* is a constitutively expressed, basal inhibitor of the pathway, *AXIN2* is a β‐catenin target gene driving inhibitory feedback regulation [11, 12, 13]. Tankyrase (TNKS) regulates the protein stability of AXIN1 and AXIN2 [14]. TNKS is a poly‐ADP‐ribosylation (PARsylation) enzyme, and directly binds to a short motive in the N‐terminus of AXIN1 and AXIN2 [14, 15]. PARsylation of AXIN1 and AXIN2 through TNKS induces consequent ubiquitination and proteasomal degradation of the two Wnt pathway inhibitors, thereby promoting Wnt signaling activity [14]. TNKS small‐molecule inhibitors block Wnt/β‐catenin signaling by stabilizing AXIN1 and AXIN2, and have therefore been investigated for targeted treatment of colorectal cancer [14, 16, 17].

In the present study, we noted that knockout or knockdown of AXIN2 in human SW480 colorectal cancer cells resulted in higher protein levels of AXIN1, which was a result of increased protein stability. Detailed quantitative analysis revealed that the increase in AXIN1 overcompensated the loss of AXIN2 with respect to protein expression levels. Yet, the loss of AXIN2 activated Wnt signaling, pointing to an AXIN2 function that could not be compensated by AXIN1. Moreover, AXIN2 loss completely disrupted inhibition of the Wnt pathway and stabilization of AXIN1 through TNKS inhibition. Our mechanistic studies with transiently expressed proteins suggest that AXIN2 promotes TNKS‐mediated degradation of AXIN1 in colorectal cancer cells by increasing TNKS–AXIN1 interaction.

## Results

### Loss of AXIN2 increases AXIN1 protein levels in colorectal cancer cells

**Fig. 1:**
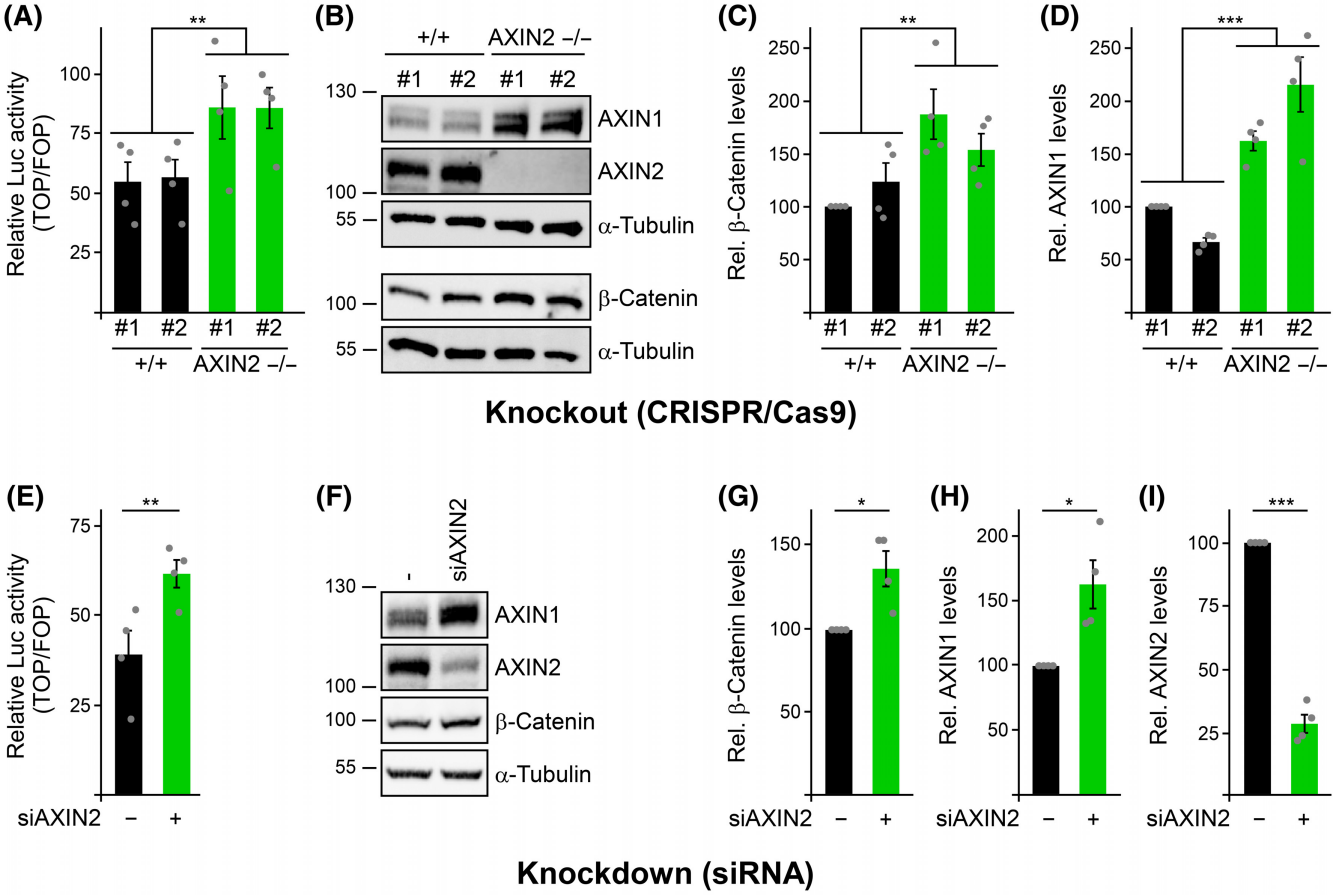
Loss of AXIN2 increases Wnt signaling and AXIN1 levels. (A–D) Analysis of parental SW480 WT cells (+/+ #1, black bars), one WT control clone (+/+ #2, black bars) and two AXIN2 knockout clones (AXIN2 −/− #1 and #2, green bars). (E–I) Analysis of SW480 cells transfected with control siRNA (−, black bars) or siAXIN2 (AXIN2 knockdown cells, green bars). (A, E) Relative luciferase activity reporting β‐catenin‐dependent transcription in the indicated cells (*n* = 4). (B, F) Western blotting for endogenous AXIN1, AXIN2, β‐catenin and α‐tubulin (loading control) in hypotonic lysates of the indicated cells. (C, D, G–I) Relative protein levels of β‐catenin (C, G), AXIN1 (D, H) or AXIN2 (I) normalized to α‐tubulin (loading control), as determined by 2D densitometry analysis of protein bands from four independent experiments [as shown in (B)] (C, D) and [as shown in (F)] (G–I; *n* = 4). Results are the mean ± SEM, *P < 0.05, **P < 0.01, ***P < 0.001 (Student's *t*‐test).

To investigate the feedback regulation of the Wnt/β‐catenin signaling pathway through AXIN2 in human colorectal cancer cells with mutant APC, we generated AXIN2 knockout clones in SW480 cells using clustered regularly interspaced short palindromic repeats (CRISPR)/CRISPR‐associated 9 (Cas9) technology. Compared to the parental SW480 cells (+/+ #1) and one wild‐type (WT) clone (+/+ #2), we observed significantly increased β‐catenin‐dependent transcription in AXIN2 knockout clones (AXIN2 −/− #1 and #2), as determined by the T cell factor optimal (TOP)‐flash luciferase reporter assay (Fig. 1A). Consistently, AXIN2 knockout clones exhibited elevated β‐catenin protein levels, demonstrating that loss of AXIN2 impairs degradation of β‐catenin in APC mutant colorectal cancer cells (Fig. 1B,C). Taken together, Wnt signaling activity was increased in AXIN2 knockout clones, in line with the loss of a negative regulator. Unexpectedly, we detected markedly increased protein levels of AXIN1 in the AXIN2 knockout clones compared to the controls (Fig. 1B,D). Although *AXIN2* is a target gene of the Wnt pathway [11], *AXIN1* is not, and thus elevated Wnt signaling could not account for the increase in AXIN1 protein levels. The increased AXIN1 levels in the AXIN2 knockout clones could be an indirect result of a growth advantage during the process of clonal selection or it could be a direct consequence of the AXIN2 loss. To discriminate between the two possibilities, we performed transient experiments with small interfering RNA (siRNA)‐mediated knockdown of AXIN2 in SW480 cells. As observed for the AXIN2 knockout clones, AXIN2 knockdown increased β‐catenin‐dependent transcription and β‐catenin protein levels (Fig. 1E–G). Importantly, AXIN2 knockdown also increased the protein levels of AXIN1 (Fig. 1F,H,I), similar to that seen after AXIN2 knockout (Fig. 1B,D). Our data show that AXIN2‐mediated feedback inhibition of the Wnt pathway is active in APC mutated colorectal cancer cells. Moreover, we discovered that AXIN2 somehow regulates the protein levels of AXIN1 in these cells because loss of AXIN2 resulted in increased protein levels of AXIN1.

### The increase of AXIN1 overcompensates the decrease of AXIN2 protein level

**Fig. 2:**
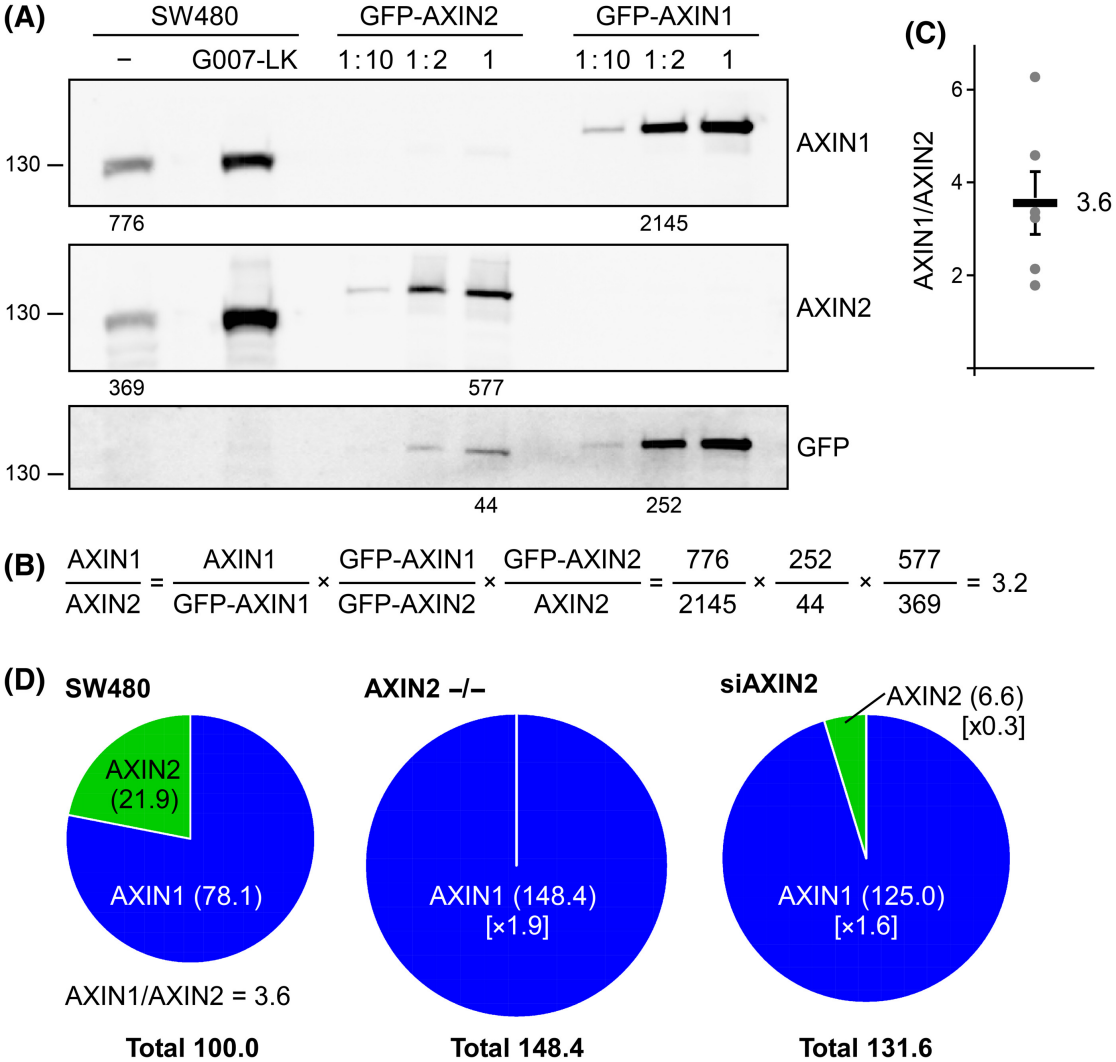
The increase in AXIN1 overcompensates for the decrease of AXIN2. (A) Western blotting for endogenous AXIN1 and AXIN2 in lysates of SW480 cells that were untreated (−) or treated with 100 nm G007‐LK overnight, and for GFP‐AXIN2 and GFP‐AXIN1 in lysates of transfected SW480 cells, which were loaded as 1 : 10 diluted, 1 : 2 diluted and undiluted, using the antibodies indicated on the right. Numbers below the blots indicate the intensities of the respective protein bands determined by 2D densitometry, and were used for the exemplary calculation in (B). (B) Calculation of the ratio of the protein level of AXIN1 relative to the protein level of AXIN2 in SW480 cells, based on the numbers determined in (A). (C) Quantification of the AXIN1/AXIN2 protein ratio in SW480 cells from six independent experiments, as shown in (A, B), as the mean ± SEM. (D) Pie charts illustrating the relative levels of AXIN1 (blue) and AXIN2 (green) in parental SW480 WT cells (SW480), SW480 AXIN2 knockout (AXIN2 −/−) and knockdown cells (siAXIN2). The sizes of the pie charts represent the combined pool of AXIN1 and AXIN2 (Total). The SW480 WT chart reflects the mean AXIN1/AXIN2 ratio of 3.6 as determined in (C), and the combined pool of AXIN1 and AXIN2 was set to 100%. The amounts of AXIN1 and AXIN2 in the AXIN2 knockout and knockdown cells were calculated trough the fold changes relative to the WT cells (provided in square brackets). These fold changes of AXIN1 in the knockout cells (mean fold change of both clones) and of AXIN1 and AXIN2 in the knockdown cells were calculated based on the data shown in Fig. 1D,H,I (for details, see Table 1).

**Fig. 3:**
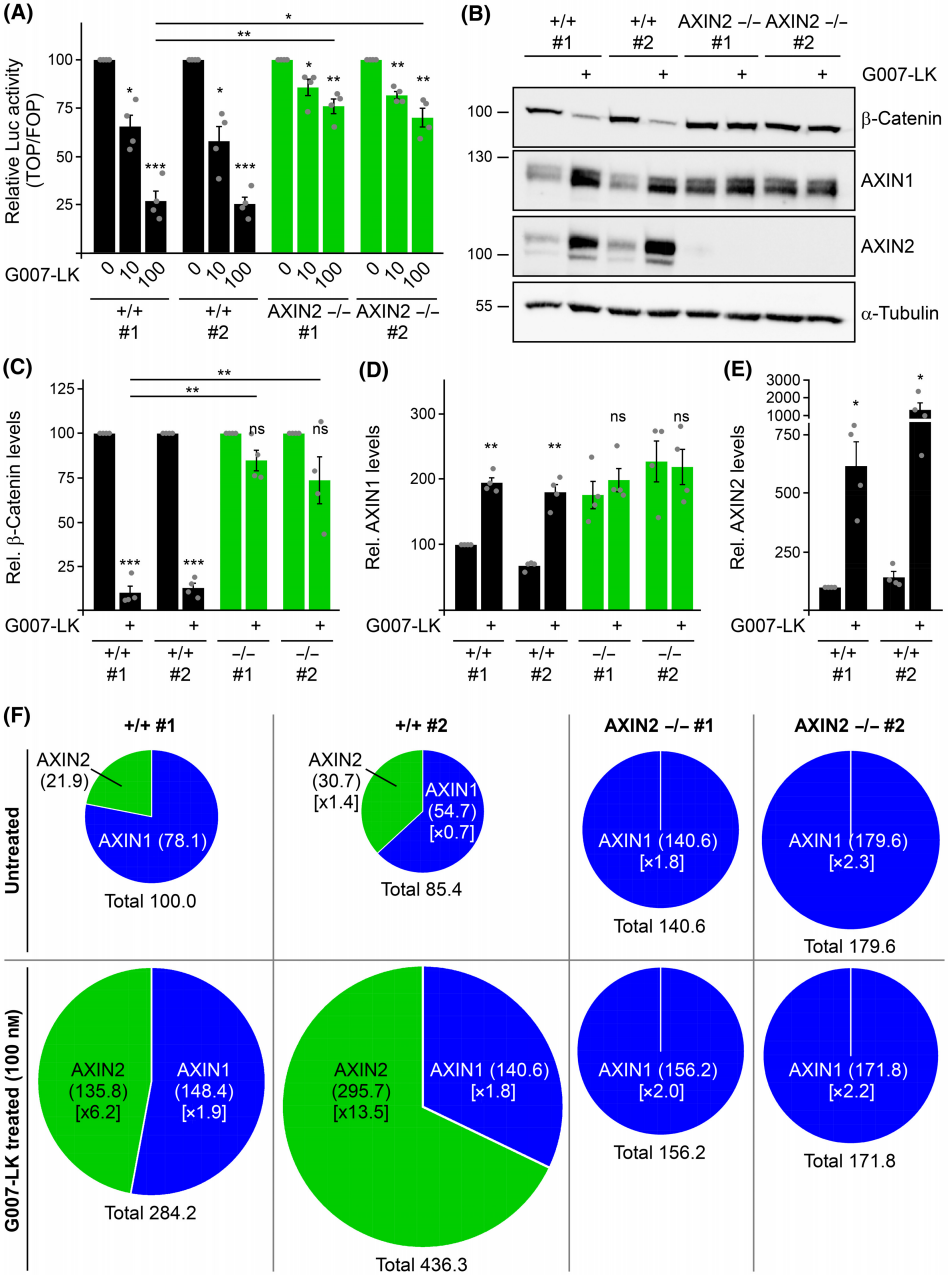
AXIN2 loss disrupts TNKS‐mediated regulation of Wnt signaling and AXIN1 levels. (A) Relative luciferase activity reporting β‐catenin‐dependent transcription in parental SW480 cells (+/+ #1, black bars), one WT control clone (+/+ #2, black bars) and two AXIN2 knockout clones (AXIN2 −/− #1 and #2, green bars), which were untreated (0) or treated with 10 or 100 nm of the TNKS small‐molecule inhibitor G007‐LK overnight, as indicated (*n* = 4). The activity in untreated cells (0) was set to 100% for every clone to facilitate comparisons of the G007‐LK effect. (B) Western blotting for endogenous β‐catenin, AXIN1, AXIN2 and α‐tubulin (loading control) in hypotonic lysates of the indicated cells without or with 100 nm G007‐LK treatment overnight (+). (C–E) Relative protein levels of β‐catenin (C), AXIN1 (D) or AXIN2 (E) normalized to α‐tubulin (loading control), as determined by 2D densitometry analysis of protein bands from four independent experiments as in (B) (*n* = 4). The β‐catenin levels (C) in untreated cells were set to 100% for every clone to facilitate comparisons of the G007‐LK effect. (A, C–E) Results are shown as the mean ± SEM, *P < 0.05, **P < 0.01, ***P < 0.001; ns (not significant), *P* > 0.05 (Student's *t*‐test). (F) Pie charts illustrating the relative levels of AXIN1 (blue) and AXIN2 (green) in the indicated cells without (untreated) or with 100 nm G007‐LK treatment overnight. The sizes of the pie charts represent the combined pool of AXIN1 and AXIN2 (Total). The parental SW480 WT chart (+/+ #1) reflects the mean AXIN1/AXIN2 ratio as determined in Fig. 2C, and the combined pool of AXIN1 and AXIN2 was set to 100%. The amounts of AXIN1 and AXIN2 in the other pie charts were calculated through the fold changes relative to the WT cells (provided in square brackets), as determined in (D) and (E) (for details of the calculation, see Table 1).

Next, we investigated how the increase of AXIN1 compares to the decrease of AXIN2 (i.e. whether the combined pool of AXIN1 and AXIN2 decreases, maintains or increases in AXIN2 knockout or knockdown cells). For this, we compared the protein levels of AXIN1 to those of AXIN2, which could not be achieved by a simple comparison of the western blot signals as a result of potential differences in the binding affinities of the primary antibodies against AXIN1 and AXIN2. Therefore, we performed an indirect comparison of AXIN1 and AXIN2 employing exogenous human green fluorescent protein (GFP)‐AXIN1 and GFP‐AXIN2 (Fig. 2A). Endogenous AXIN1 was compared with GFP‐AXIN1 using the α‐AXIN1 antibody; GFP‐AXIN1 was compared with GFP‐AXIN2 using the α‐GFP antibody; and GFP‐AXIN2 was compared to endogenous AXIN2 using the α‐AXIN2 antibody, allowing comparison of the protein levels of endogenous AXIN1 with those of AXIN2 (Fig. 2A,B). Several repetitions of this analysis determined a ratio of AXIN1 to AXIN2 protein levels in the parental SW480 WT cells of approximately 3.6 (Fig. 2C), meaning that the combined pool of AXIN1 and AXIN2 consists of 78.1% AXIN1 and 21.9% AXIN2 (Fig. 2D). Inhibiting TNKS‐mediated degradation of AXIN1 and AXIN2 through G007‐LK treatment appeared to alter this ratio because AXIN2 was stabilized more prominently than AXIN1, which is analyzed in detail below (Fig. 3). In the AXIN2 knockout clones, the AXIN2 protein levels were lost (0%) and the AXIN1 levels increased on average by 1.9‐fold (78.1% × 1.9 = 148.3%, based on the data of Fig. 1D) compared to the parental cells. Thus, the combined pool of AXIN1 and AXIN2 increased in the AXIN2 −/− clones (0% AXIN2 + 148.3% AXIN1 = 148.3%) compared to the WT control cells (Fig. 2D). Upon siRNA‐mediated AXIN2 knockdown, the AXIN2 protein levels decreased to 0.3‐fold (21.9% × 0.3 = 6.6%, based on the data of Fig. 1I) and the AXIN1 levels increased by 1.6‐fold (78.1% × 1.6 = 124.9%, based on the data of Fig. 1H) compared to the control cells. Thus, the combined pool of AXIN1 and AXIN2 increased in the AXIN2 knockdown cells (6.6% AXIN2 + 124.9% AXIN1 = 131.5%) compared to the control cells (Fig. 2D). In summary, the analysis revealed that the increase of AXIN1 protein quantitatively overcompensated the decrease of AXIN2 because the combined pool of AXIN1 and AXIN2 increased from 100% in WT cells to approximately 150% in AXIN2 knockout cells and to approximately 130% in AXIN2 knockdown cells (Fig. 2D). However, the expression level overcompensating increase of AXIN1 failed to rescue the decrease of AXIN2 functionally because knockout or knockdown of AXIN2 activated Wnt signaling (Fig. 1A,E), suggesting that AXIN2 is crucial for Wnt pathway regulation in colorectal cancer cells.

### Loss of AXIN2 disrupts regulation of Wnt signaling and of AXIN1 levels through TNKS

**Fig. 4:**
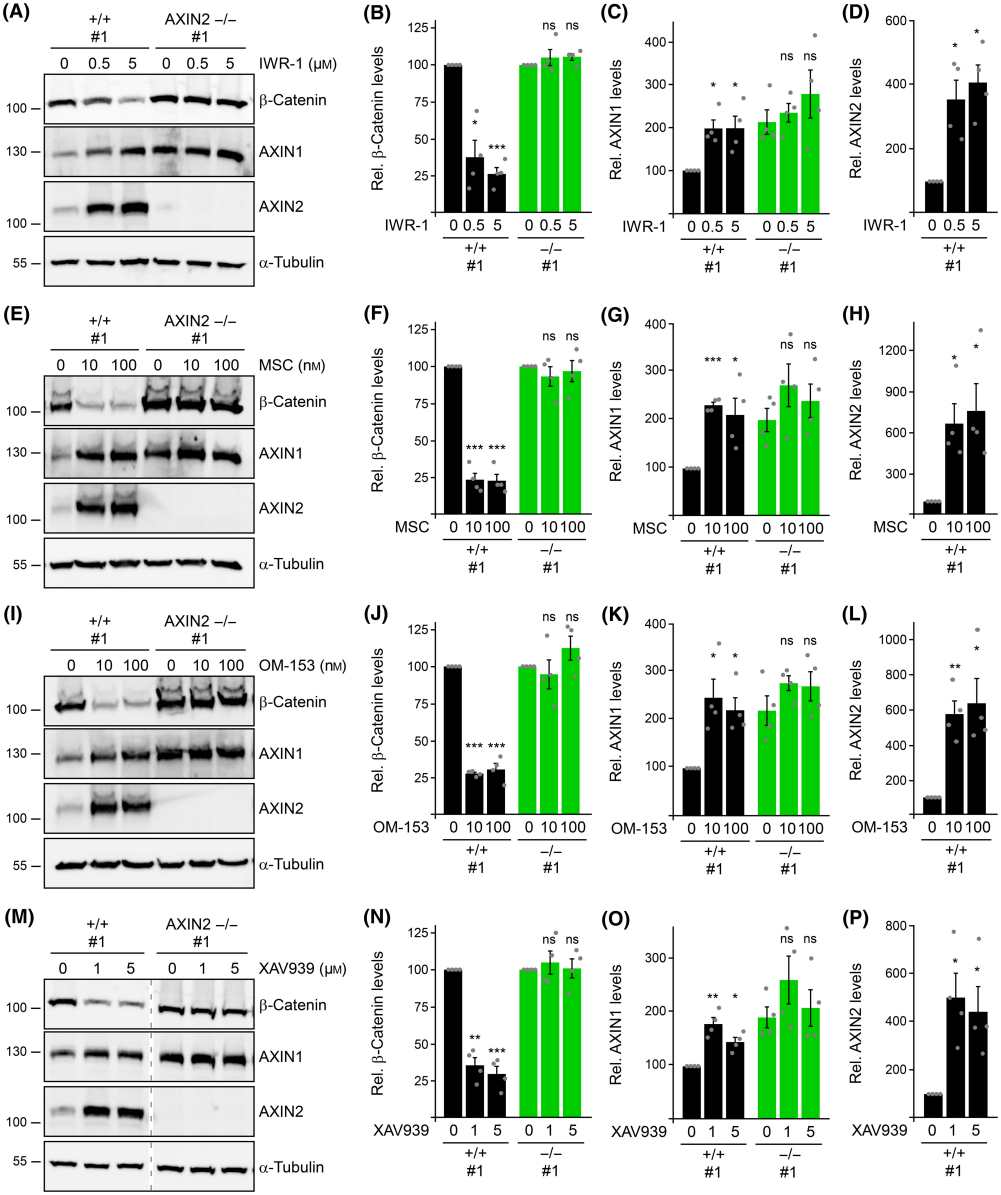
TNKS inhibitors depend on AXIN2 for regulating β‐catenin and AXIN1 levels. (A, E, I, M) Western blotting for endogenous β‐catenin, AXIN1, AXIN2 and α‐tubulin (loading control) in lysates of SW480 WT cells (+/+) and AXIN2 knockout cells (−/−), which were untreated or treated with TNKS small‐molecule inhibitors (IWR‐1, MSC2504877, OM‐153, XAV939) overnight, as indicated. Dotted vertical lines in (M) indicate splicing of the blots. (B–D, F–H, J–L, N–P) Relative protein levels of β‐catenin (B, F, J, N), AXIN1 (C, G, K, O) or AXIN2 (D, H, L, P) normalized to α‐tubulin (loading control), as determined by 2D densitometry analysis of protein bands from four independent experiments as in (A), (E), (I) and (M), respectively (*n* = 4). The β‐catenin levels (B, F, J, N) in untreated cells were set to 100% for WT and knockout cells to facilitate comparisons of the TNKS inhibitor effect. Results are shown as the mean ± SEM, *P < 0.05, **P < 0.01, ***P < 0.001; ns (not significant), *P* > 0.05 (Student's *t*‐test).

TNKS mediates degradation of AXIN1 and AXIN2, and inhibition of TNKS causes stabilization of the two negative Wnt pathway regulators and consequent silencing of the pathway [14]. Because our data pointed to an essential role for AXIN2 in regulating the Wnt pathway in colorectal cancer cells, we investigated the particular relevance of AXIN2 for Wnt pathway inhibition by TNKS small‐molecule inhibitors in these cells. In SW480 WT control cells, TNKS inhibition by G007‐LK strongly inhibited β‐catenin‐dependent transcription in a dosage‐dependent manner (Fig. 3A). Consistently, G007‐LK treatment markedly increased degradation of β‐catenin in these cells (Fig. 3B,C). By contrast, in AXIN2 knockout cells, G007‐LK treatment inhibited Wnt signaling very weakly and failed to promote degradation of β‐catenin (Fig. 3A–C). In the WT control cells, TNKS inhibition stabilized AXIN1 by approximately two‐fold (Fig. 3B,D), whereas AXIN2 was stabilized between six‐ and 10‐fold (Fig. 3B,E), in line with previous observations [14, 18]. The lack of this strong AXIN2 increase could potentially explain why TNKS inhibition did not promote degradation of β‐catenin in the AXIN2 knockout cells. Unexpectedly, we noted that TNKS inhibition also completely failed to further stabilize AXIN1 in the AXIN2 knockout cells (Fig. 3B,D). For a more complete picture, we calculated the combined pools of AXIN1 and AXIN2 for the parental SW480 cells (+/+ #1), for one WT control clone (+/+ #2) and for two AXIN2 knockout clones (AXIN2 −/− #1 and #2) without and with TNKS inhibition (Fig. 3F). The calculation is based on the previously determined ration between AXIN1 and AXIN2 in the parental SW480 cells (Fig. 2D) and the fold changes of AXIN1 and AXIN2 in the respective clones without and with TNKS inhibition relative to the parental cells (Fig. 3D,E; for details of the calculation, see also Table 1). This analysis showed that the combined pool of AXIN1 and AXIN2 increased between approximately three‐ to five‐fold in the WT cells (Fig. 3F). Within the combined pool, the proportion of AXIN2 markedly increased upon TNKS inhibition (Fig. 3F) because of the much stronger increase of AXIN2 compared to AXIN1 (Fig. 3D,E), suggesting that AXIN2 gains additional importance for Wnt pathway inhibition under this condition. By contrast, in the AXIN2 knockout clones, TNKS inhibition did not alter the combined pool of AXIN1 and AXIN2 at all (Fig. 3F). Similarly as observed with G007‐LK (Fig. 3B–D), other small‐molecule TNKS inhibitors, such as IWR‐1 (Fig. 4A–D), MSC2504877 (Fig. 4E–H), OM‐153 (Fig. 4I–L) and XAV939 (Fig. 4M–P), failed to induce degradation of β‐catenin and stabilization of AXIN1 in AXIN2 knockout cells. Our experiments revealed that inhibition of Wnt signaling by TNKS small‐molecule inhibitors in SW480 colorectal cancer cells strictly depends on AXIN2.

**Table 1:** Calculation of the pie charts. Row 1: headings. Row 2: percentages of AXIN2 (21.9) and AXIN1 (78.1) in untreated SW480 WT cells (left pie chart in Fig. 2D) were calculated based on the ratio of AXIN2: AXIN1 of 1: 3.56, as determined in Fig. 2C (*n* = 6). Rows 3 to 12: for all other cell lines and/or treatment conditions (indicated in the second column), the percentages of AXIN2 and AXIN1 were calculated using the fold changes relative to the untreated SW480 WT cells, as shown in the fourth and fifth column for AXIN2 and AXIN1, respectively. The fold changes of AXIN2 and AXIN1 relative to WT were calculated as shown in the sixth and seventh column, respectively, and are based on the indicated figures. For the fold change calculations, means of four independent biological replicates were used (*n* = 4).

Fig.	Cells and treatment	Total	AXIN2	AXIN1	AXIN2 fold relative to WT	AXIN1 fold relative to WT
2D	SW480	**100**	**21.9** = 100/(1 + 3.56) × 1, based on Fig. 2C; *n* = 6	**78.1** = 100/(1 + 3.56) × 3.56, based on Fig. 2C; *n* = 6	NA	NA
2D	AXIN2−/−	**148.4** = 0 + 148.4	**0**	**148.4** = 78.1 × 1.9	NA	= [(161.9 + 214.9): 2]: 100 = **1.9** = [(AXIN2−/−#1 + AXIN2−/−#2): 2]: +/+#1, based on Fig. 1D; *n* = 4
2D	siAXIN2	**131.6** = 6.6 + 125.0	**6.6** = 21.9 × 0.3	**125.0** = 78.1 × 1.6	= 28.7: 100 = **0.3** = siAXIN2+: siAXIN2−, based on Fig. 1I; *n* = 4	= 163.2: 100 = **1.6** = siAXIN2+: siAXIN2−, based on Fig. 1H; *n* = 4
3F	+/+ #1 ut	= SW480 Fig. 2D	= SW480 Fig. 2D	= SW480 Fig. 2D	NA	NA
3F	+/+ #1 G007‐LK	**284.2** = 135.8 + 148.4	**135.8** = 21.9 × 6.2	**148.4** = 78.1 × 1.9	= 615.4: 100 = **6.2** = +/+#1G007‐LK: +/+#1ut, based on Fig. 3E; *n* = 4	= 194.2: 100 = **1.9** = +/+#1G007‐LK: +/+#1ut, based on Fig. 3D; *n* = 4
3F	+/+ #2 ut	**85.4** = 30.7 + 54.7	**30.7** = 21.9 × 1.4	**54.7** = 78.1 × 0.7	= 141.2: 100 = **1.4** = +/+#2ut: +/+#1ut, based on Fig. 3E; *n* = 4	= 67.9: 100 = **0.7** = +/+#2ut: +/+#1ut, based on Fig. 3D; *n* = 4
3F	+/+ #2 G007‐LK	**436.3** = 295.7 + 140.6	**295.7** = 21.9 × 13.5	**140.6** = 78.1 × 1.8	= 1353.8: 100 = **13.5** = +/+#2G007‐LK: +/+#1ut, based on Fig. 3E; *n* = 4	= 179.9: 100 = **1.8** = +/+#2G007‐LK: +/+#1ut, based on Fig. 3D; *n* = 4
3F	AXIN2 −/− #1 ut	**140.6** = 0 + 140.6	**0**	**140.6** = 78.1 × 1.8	NA	= 175.7: 100 = **1.8** = −/−#1ut: +/+#1ut, based on Fig. 3D; *n* = 4
3F	AXIN2 −/− #1 G007‐LK	**156.2** = 0 + 156.2	**0**	**156.2** = 78.1 × 2.0	NA	= 198.3: 100 = **2.0** = −/−#1G007‐LK: +/+#1ut, based on Fig. 3D; *n* = 4
3F	AXIN2 −/− #2 ut	**179.6** = 0 + 179.6	**0**	**179.6** = 78.1 × 2.3	NA	= 226.9: 100 = **2.3** = −/−#2ut: +/+#1ut, based on Fig. 3D; *n* = 4
3F	AXIN2 −/− #2 G007‐LK	**171.8** = 0 + 171.8	**0**	**171.8** = 78.1 × 2.2	NA	= 218.4: 100 = **2.2** = −/−#2G007‐LK: +/+#1ut, based on Fig. 3D; *n* = 4

### AXIN2 promotes degradation of AXIN1 through TNKS

**Fig. 5:**
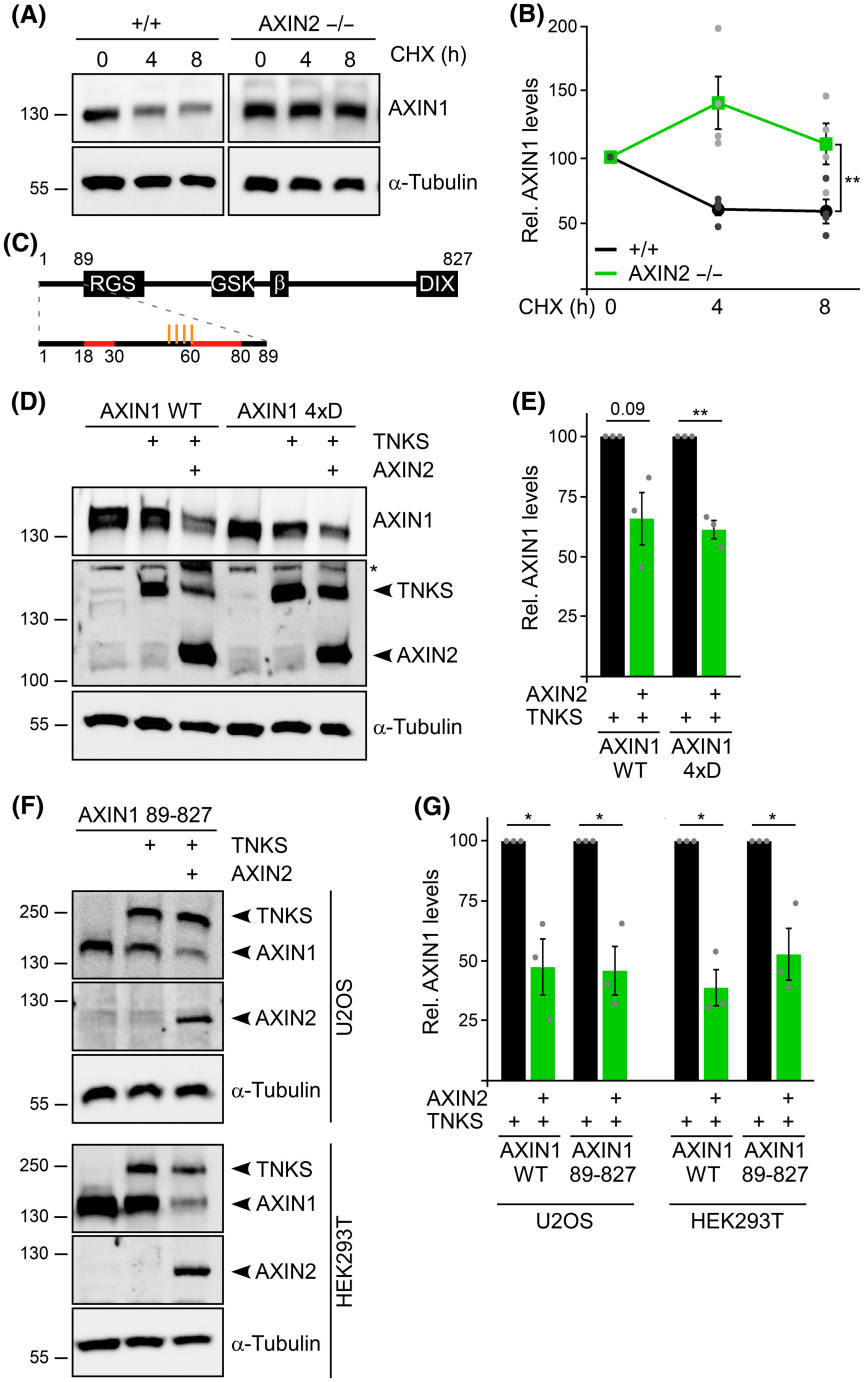
AXIN2 promotes degradation of AXIN1. (A) Western blotting for endogenous AXIN1 and α‐tubulin (loading control) in lysates of parental SW480 WT cells (+/+) and AXIN2 knockout cells (−/−), which were untreated (0) or treated with 100 μm CHX for 4 or 8 h. (B) Relative protein levels of AXIN1 normalized to α‐tubulin (loading control), as determined by 2D densitometry analysis of protein bands from four independent experiments as in (A). (C) Schematic to scale representation of rat AXIN1 WT (1–827) with its binding sites for APC (RGS), GSK3B (GSK), β‐catenin (β) and the polymerizing DIX domain. Amino acids 1–89 are magnified below, and the two TNKS binding segments [15] and four phosphorylation sites that interfere with TNKS‐mediated degradation of AXIN1 [19] are highlighted in red and orange, respectively. (D, F) Western blotting for GFP‐AXIN1 WT and 4xD, Flag‐TNKS and Flag‐AXIN2 in lysates of U2OS cells (D), or for GFP‐AXIN1 89‐827, GFP‐TNKS and Flag‐AXIN2 in lysates of U2OS and HEK293T cells (F), as indicated. Cells have been transiently transfected as indicated above the blots. α‐Tubulin: loading control. Asterisk: unspecific band. (E, G) Relative protein levels of AXIN1 WT and 4xD (E) and of AXIN1 WT and 89–827 (G) normalized to α‐tubulin (loading control) in cells co‐expressing TNKS without or with additional co‐expression of AXIN2, as determined by 2D densitometry analysis of protein bands from three independent experiments (*n* = 3) [as shown in (D)] (E) and [as shown in (F)] (G). (B, E, G) Results are shown as the mean ± SEM, *P < 0.05, **P < 0.01 (Student's *t*‐test).

**Fig. 6:**
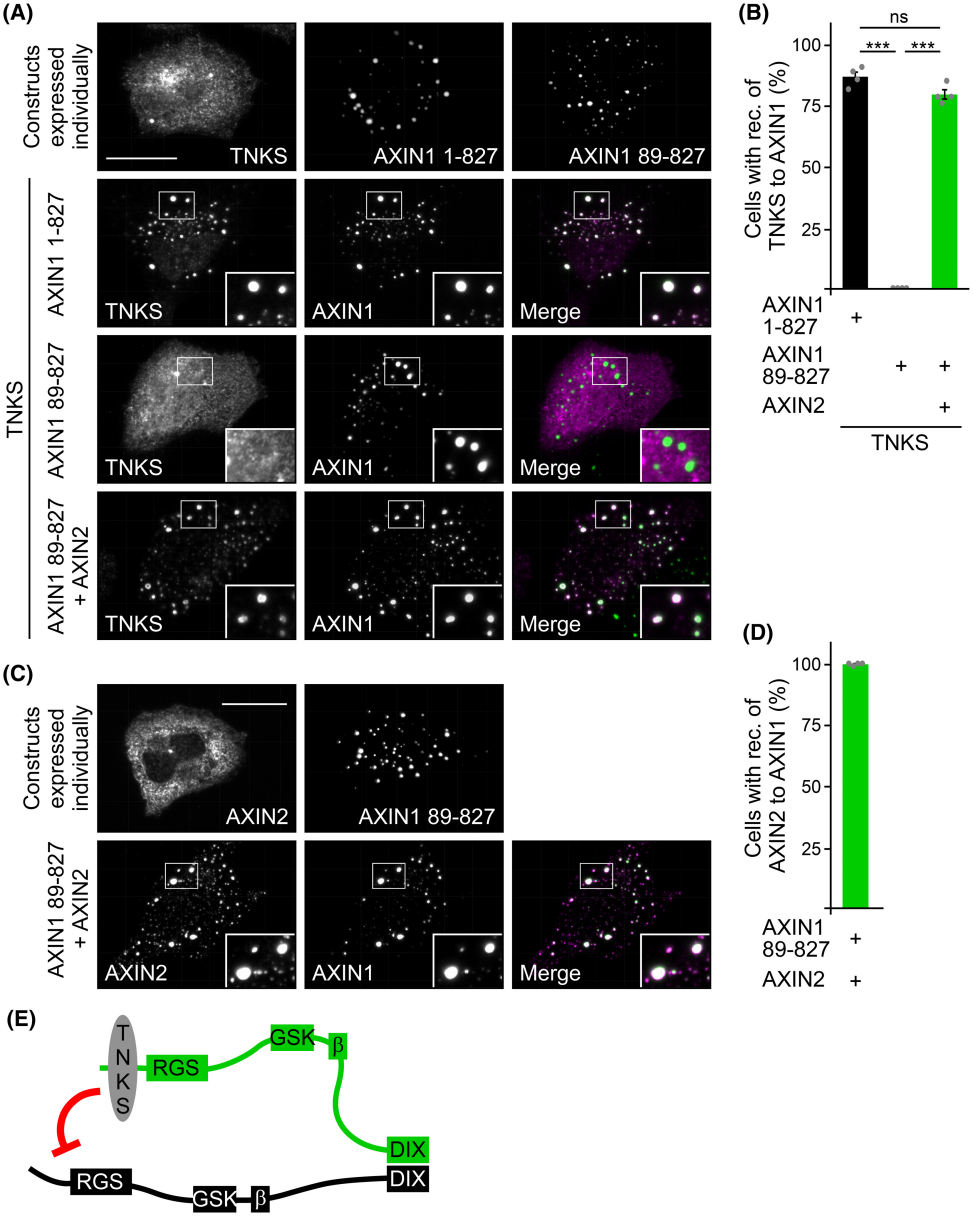
AXIN2 recruits TNKS to AXIN1. (A, C) Immunofluorescence staining (magenta) of Flag‐TNKS (A) or HA‐AXIN2 (C) and GFP fluorescence (green) in U2OS cells, which were transfected with Flag‐TNKS, GFP‐AXIN1 1‐827 (WT), GFP‐AXIN1 89‐827 and HA‐AXIN2, either individually or in combinations, as indicated on the left. Insets are magnifications of the boxed areas. Scale bars = 20 μm. (B, D) Percentage of cells exhibiting recruitment (rec.) of TNKS (B) or AXIN2 (D) to the indicated AXIN1 proteins out of 332–356 cells from 200 merged images of four independent experiments (*n* = 4) [as shown in (A)] (B) and [as shown in (C)] (D). Results are the mean ± SEM, ***P < 0.001 (Student's *t*‐test). (E) Schematic representation of AXIN1 (black), AXIN2 (green) and TNKS (gray), illustrating how the DIX‐DIX interaction between AXIN1 and AXIN2 promotes recruitment of AXIN2‐bound TNKS to AXIN1, facilitating degradation of AXIN1 through TNKS (red blocking arrow). Binding sites for APC (RGS), GSK3B (GSK), β‐catenin (β) and the polymerizing DIX domain are indicated.

We observed increased protein levels of AXIN1 in the AXIN2 knockout clones compared to the controls (Fig. 1B). Because the increased Wnt signaling activity in the AXIN2 knockout clones would not stimulate transcription of AXIN1, it was likely that the AXIN1 protein was more stable in the AXIN2 knockout clones. To test this hypothesis, we monitored the stability of the AXIN1 protein after inhibition of protein translation by cycloheximide. Although approximately 50% of AXIN1 were degraded after 8 h of cycloheximide treatment in the SW480 WT cells, almost no degradation occurred in the AXIN2 knockout cells, demonstrating that AXIN1 was significantly more stable in the absence of AXIN2 (Fig. 5A,B). The AXIN1 levels in G007‐LK‐treated WT cells were intriguingly similar to those in untreated AXIN2 knockout cells, and G007‐LK treatment failed to further increase the levels in the knockout cells (Fig. 3D), suggesting that loss of AXIN2 disrupted degradation of AXIN1 through TNKS. Therefore, we analyzed whether AXIN2 promotes TNKS‐mediated degradation of AXIN1. Degradation of AXIN1 through TNKS can be investigated by transient co‐expression of both proteins [19], and we used expression levels at which only weak degradation of AXIN1 occurred (Fig. 5D). Indeed, additional co‐expression of AXIN2 markedly enhanced degradation of AXIN1 through TNKS in this assay (Fig. 5D,E,G). We reported previously that phosphorylation of AXIN1 at residues within the TNKS binding site (Fig. 5C, orange and red marks, respectively) impairs degradation through TNKS [19]. Notably, co‐expression of AXIN2 also significantly enhanced degradation of a respective phospho‐mimicking AXIN1 mutant (AXIN1 4xD) through TNKS (Fig. 5D,E). Moreover, AXIN2 even enhanced TNKS‐mediated degradation of an AXIN1 deletion mutant (AXIN1 89‐827) that lacks the TNKS binding site (Fig. 5C,F,G). Because AXIN2 interacts with TNKS via its N‐terminal TNKS binding site and with AXIN1 via the C‐terminal DIX domain, we tested whether AXIN2 links AXIN1 to TNKS using a microscopy assay. Individually expressed TNKS showed diffuse cellular distribution with some spherical structures, and co‐expressed WT AXIN1 (AXIN1 1‐827) efficiently recruited TNKS into the typical AXIN1 condensates [8, 9], resulting in co‐localization of the two proteins (Fig. 6A,B). The AXIN1 deletion mutant lacking the TNKS binding site (AXIN1 89‐827, Fig. 5C) formed condensates, similar to WT AXIN1 (Fig. 6A). However, AXIN1 89‐827 completely failed to recruit TNKS to its condensates, demonstrating that co‐localization of the two proteins strictly depends on AXIN1–TNKS binding (Fig. 6A,B). Importantly, additional co‐expression of AXIN2 strongly promoted recruitment of TNKS in the AXIN1 89‐827 condensates, completely rescuing the lack of the TNKS binding site in AXIN1 89‐827 (Fig. 6A,B). Because AXIN2 was highly recruited to AXIN1 89‐827 condensates itself, it may directly link AXIN1 to TNKS (Fig. 6C,D). Our combined data suggest that AXIN2 promotes TNKS‐mediated degradation of AXIN1 by linking AXIN1 to TNKS, potentially explaining the increased protein stability of AXIN1 in SW480 AXIN2 knockout cells.

## Discussion

Wnt/β‐catenin signaling is pathologically hyperactive in more than 90% of the colorectal cancer cases [4]. Most frequently, inactivating mutations of the tumor suppressor APC impair degradation of β‐catenin, resulting in higher β‐catenin‐dependent transcription, and initiating tumor growth [4, 20]. *AXIN2* is highly expressed in colorectal cancer because it is a β‐catenin target gene [11]. However, AXIN2‐mediated feedback inhibition of the pathway obviously fails to restrict the Wnt signaling activity to sub‐oncogenic levels. To investigate the activity of the AXIN2 feedback in APC‐mutated colorectal cancer in a clean system, we generated SW480 AXIN2 knockout cells using the CRISPR/Cas9 technique. Importantly, β‐catenin levels and β‐catenin‐dependent transcription were elevated by approximately 50% in AXIN2 knockout cells compared to WT control cells (Fig. 1A–C). Thus, AXIN2 restricts Wnt signaling to some extent even in APC mutated colorectal cancer cells. Unexpectedly, we observed that knockout of AXIN2 markedly increased the protein levels of AXIN1 (Fig. 1B). A detailed quantitative comparison between AXIN1 and AXIN2 protein levels in SW480 cells revealed that the increase of AXIN1 overcompensated the loss of AXIN2, meaning that the amount of AXIN1 in the AXIN2 knockout cells was higher than the combined amounts of AXIN1 and AXIN2 in the control cells (Fig. 2). Yet, Wnt signaling activity and β‐catenin levels were increased in the AXIN2 knockout cells (Fig. 1A–C). Thus, the overcompensating increase in AXIN1 protein did not functionally compensate for β‐catenin degradation by AXIN2, pointing to a special importance of AXIN2 for Wnt pathway regulation in colorectal cancer cells. We showed previously that AXIN2 is less well inhibited by upstream Wnt signaling than AXIN1 [7]. Because pathway activation by Wnt ligands plays a role in colorectal cancer, the reduced inhibitability of AXIN2 compared to AXIN1 could potentially explain its special importance. Moreover, we demonstrated that it is sufficient to enhance the activity of AXIN2 to inhibit Wnt signaling in colorectal cancer in spite of the rather small amount of AXIN2 compared to AXIN1 [10, 21], which is consistent with a high importance of AXIN2 for regulating Wnt signaling in cancer cells.

The increase of AXIN1 in the AXIN2 knockout cells was interesting because, in contrast to *AXIN2*, *AXIN1* is no β‐catenin target gene, and thus elevated β‐catenin‐dependent transcription could not explain this observation. Indeed, our cycloheximide experiments showed that the AXIN1 protein is more stable in AXIN2 knockout cells than in WT control cells (Fig. 5A,B), suggesting that AXIN2 promotes degradation of AXIN1. The protein stability of AXIN1 and AXIN2 is regulated by TNKS. TNKS directly binds to AXIN1 and AXIN2, and PARsylates these proteins, thereby earmarking them for subsequent ubiquitination and proteasomal degradation [14]. Experiments with transiently expressed proteins revealed that AXIN2 promoted TNKS‐mediated degradation of WT AXIN1 as well as of AXIN1 mutants with impaired TNKS binding in different cell lines (Fig. 5D–F). Mechanistically, AXIN2 most likely enhanced the processivity of AXIN1 through recruiting TNKS to AXIN1 (Fig. 6) because AXIN2 directly binds to both TNKS and AXIN1 via an N‐terminal binding motive and via the C‐terminal DIX domain, respectively. In SW480 WT cells, TNKS inhibition stabilized AXIN2 approximately 3.5‐fold more than AXIN1 (AXIN2: eight‐fold versus AXIN1 2.3‐fold; Fig. 3D,E, mean of +/+ #1 and +/+ #2), suggesting a higher TNKS‐mediated turnover of AXIN2 compared to AXIN1. Therefore, it is feasible that binding of AXIN2 to AXIN1 increases the TNKS‐mediated turnover of AXIN1, as suggested by our proposed mechanism. We recently discovered a phosphorylation in AXIN1 impairing TNKS binding and TNKS‐mediated degradation, which is not conserved in AXIN2, probably contributing to the reduced processivity of AXIN1 by TNKS [19]. Notably, AXIN2 was able to promote TNKS‐mediated degradation of a respective phospho‐mimicking AXIN1 mutant (Fig. 5D,E). Our proposed mechanism of AXIN2‐induced degradation of AXIN1 will correlate with the expression levels of AXIN2 and therefore will be most active in colorectal cancer cells expressing high AXIN2 levels [11]. However, AXIN2‐induced degradation of AXIN1 might also play a role under physiologic conditions by inducing a switch from AXIN1‐based β‐catenin destruction complexes in unstimulated cells to AXIN2‐based β‐catenin destruction complexes in Wnt‐stimulated cells, which will start to express AXIN2 as feedback regulator [11]. According to our previous findings noted above [7], the AXIN2‐based β‐catenin destruction complexes will remain more active in the presence of Wnt ligands than those based on AXIN1, allowing effective feedback regulation.

Inhibition of TNKS through TNKS small‐molecule inhibitors stabilizes AXIN1 and AXIN2 by preventing TNKS‐mediated degradation, and consequently inhibits Wnt/β‐catenin signaling [14]. Therefore, such substances have been developed and investigated as agents for targeted therapy of colorectal cancers driven by pathologically elevated β‐catenin signaling [16, 17]. In the present study, we noted that TNKS inhibition through the broadly used TNKS inhibitor G007‐LK failed to inhibit Wnt/β‐catenin signaling in AXIN2 knockout colorectal cancer cells. In line with our finding, Thorvaldsen *et al*. [18] reported that AXIN2 plays a predominant role over AXIN1 in degradation of β‐catenin upon TNKS inhibition, based on siRNA knockdown experiments. Our even more striking differences might be a result of the more clean system of AXIN2 knockout compared to siRNA‐mediated knockdown. As discussed above, inhibition of TNKS stabilized AXIN2 much more potently than AXIN1 (Fig. 3D,E), consistent with previous studies [14, 18]. The loss of the strong stabilization of AXIN2 in the AXIN2 knockout cells could already potentially explain why TNKS inhibition failed to inhibit Wnt signaling in these cells. However, in addition, we found that the higher basal AXIN1 levels in the AXIN2 knockout cells did not further increase upon inhibition of TNKS (Fig. 3D). Thus, TNKS inhibition did not increase any AXIN protein levels in the AXIN2 knockout cells (Fig. 3F), explaining why it failed to inhibit Wnt signaling. The missing stabilization of AXIN1 upon TNKS inhibition in the AXIN2 knockout cells suggests that loss of AXIN2 disrupts degradation of AXIN1 by TNKS, supporting our proposed model that AXIN2 promotes TNKS‐mediated degradation of AXIN1. Moreover, the high AXIN2 dependency of Wnt pathway inhibition by TNKS inhibitors in colorectal cancer cells implies that TNKS inhibitors will never block Wnt signaling completely because this would prevent transcription of the β‐catenin target gene *AXIN2*, causing AXIN2 depletion. Alternatively, Wnt pathway inhibition by TNKS inhibitors might follow an oscillating pattern, allowing phases with higher signaling activity to regenerate AXIN2. These considerations might become relevant for future colorectal cancer therapy by TNKS small‐molecule inhibitors.

## Materials and methods

### Cell culture, transfection and treatments

In the present study, human SW480 (RRID: CVCL_0546) colorectal cancer cells, U2OS (RRID: CVCL_0042) cells and HEK293T (RRID: CVCL_0063) cells were used, which had been originally obtained from ATCC (Manassas, VA, USA). The cells were cultured in low‐glucose Dulbecco's modified Eagle's medium (Thermo Fisher Scientific, Waltham, MA, USA) at 37 °C in a 10% CO_2_ atmosphere, and passaged according to ATCC recommendations. Cells were authenticated based on cell morphology, cell size and indicative features (e.g. truncated APC in SW480 cells) and were tested negative for mycoplasma contamination. Lipofectamine 2000 (SW480 cells; Thermo Fisher Scientific) and polyethylenimine (U2OS and HEK293T cells) were used for transfection of plasmid DNA, and Oligofectamine (Thermo Fisher Scientific) was used for transfection of siRNA, in accordance with the manufacturer's instructions. TNKS small‐molecule inhibitors G007‐LK, IWR‐1, XAV939 (Merck, Darmstadt, Germany), MSC2504877 and OM‐153 (Biozol Diagnostics, Eching, Germany), as well as the inhibitor of protein translation cycloheximide (CHX; Merck), were used for cell treatments. The applied concentrations of the inhibitors and the treatment times of individual experiments are provided where appropriate.

### SW480 CRISPR/Cas9 AXIN2 knockout cells

Generation of the SW480 CRISPR/Cas9 knockout cells has been described previously [10]. A guide RNA sequence (CGAGATCCAGTCGGTGATGG) targeting the first coding exon of the human *AXIN2* gene was inserted into the PX458 vector (#48138; Addgene, Watertown, MA, USA) [22] additionally encoding for the Cas9 enzyme and GFP, using standard molecular biology methods. SW480 cells were transfected with the generated vector, and positively transfected cells were sorted according to the GFP expression and seeded as one cell per well in 96‐well plates. The outgrowing cell clones were screened for loss of AXIN2 expression via western blotting against AXIN2 and α‐tubulin (loading control) (see below). Afterwards, the successful editing of the *AXIN2* gene via CRISPR/Cas9 was confirmed by sequencing. As controls, clones with unaltered AXIN2 expression were chosen, and the WT sequence of the *AXIN2* gene was verified by sequencing.

### Molecular biology

AXIN1, AXIN2 and TNKS that constructs were conjugated to GFP, the Flag‐tag or the HA‐tag were: GFP‐AXIN1 (human), GFP‐AXIN1 (rat), GFP‐AXIN1 89‐827 (rat), GFP‐AXIN2 (human), Flag‐AXIN2 (Flag‐Conductin, mouse), HA‐AXIN2 (HA‐Conductin, mouse), GFP‐TNKS (human) and Flag‐TNKS (human), as described previously [10, 19]. GFP‐AXIN1 4xD (S51D, S54D, S57D, T60D, rat) was generated via site‐directed mutagenesis and verified by sequencing.

### Antibodies and siRNAs

Primary antibodies: rb α AXIN1 [western blotting (WB): dilution 1 : 1000], 2087S; rb α AXIN2 (WB: dilution 1 : 1000), 2151S (Cell Signaling Technology, Danvers, MA, USA)/m α β‐catenin (WB: dilution 1 : 1000), sc‐7963 (Santa Cruz Biotechnologies, Dallas, TX, USA)/rat α α‐tubulin (WB: dilution 1 : 1000), MCA77G (Serotec; Bio‐Rad, Hercules, CA, USA)/m α GFP (WB: dilution 1 : 1000), 11814460001; rb α Flag [WB: dilution 1 : 1000, immunofluorescence (IF): dilution 1 : 400], F7425; and rb α HA (IF: dilution 1 : 200), H6908 (Merck). Antibodies against endogenous proteins (α AXIN1, α AXIN2, α‐catenin and α β‐catenin) were validated at our laboratory by knockdown and/or knockout experiments. Antibodies against tags (α GFP, α Flag and α HA) were validated by the absence of signal without transient expression of tagged proteins.

Secondary antibodies: goat α mouse/rabbit/rat‐horseradish peroxidase (HRP) [dilution 1 : 1000 to dilution 1 : 2000], goat α rabbit‐Cy3 [dilution 1 : 300] (Bio‐Rad).

The siRNA targeting human AXIN2 (5′‐GAGAUGGCAUCAAGAAGCA‐3′) has been used previously [10].

### Luciferase reporter assay

To measure the transcriptional activity of β‐catenin via a luciferase reporter, cells were transfected with a plasmid encoding for the firefly luciferase under the control of a β‐catenin‐sensitive promoter (TOP), together with a constitutive β‐galactosidase expression plasmid. By contrast, control cells were transfected with a mutated, β‐catenin‐insensitive luciferase plasmid (Far from optimal, FOP). The cells were lysed approximately 24 h after transfection in a buffer preserving luciferase and β‐galactosidase enzymatic activity (25 mm Tris‐HCl, pH 8, 2 mm EDTA, 5% glycerol, 1% Triton X‐100, 20 mm dithiothreitol). From these lysates, the luciferase activity was measured via light emission upon luciferin decarboxylation in a Centro LB 960 Microplate Luminometer (Berthold Technologies, Bad Wildbad, Germany). β‐galactosidase activity was measured as release of yellow ortho‐nitrophenol upon ortho‐nitrophenyl‐β‐galactoside hydrolysis using a Spectra MAX 190 (Molecular Devices, San Jose, CA, USA). Finally, TOP luciferase activity and the FOP luciferase activity were normalized to the respective β‐galactosidase activities to correct for transfection variances, before calculating the normalized TOP/FOP ratio reflecting β‐catenin‐dependent transcription.

### Western blotting

Cells, which were transfected and/or treated, as indicated where appropriate, were lysed in Triton X‐100‐containg lysis buffer (25 mm Tris‐HCl, pH 8, 2 mm EDTA, 5% glycerol, 1% Triton X‐100, 20 mm dithiothreitol, Roche protease inhibitor cocktail) or in hypotonic lysis buffer (20 mm Tris‐HCl, pH 7.5, 1 mm EDTA, Roche protease inhibitor cocktail) for assessing β‐catenin levels (Figs 1B,F and 3B). The cell extracts were denatured and subjected to SDS/PAGE, and the size‐separated proteins were blotted onto a nitrocellulose membrane (VWR International, Radnor, PA, USA). The membranes were probed with indicated primary and respective HRP‐conjugated secondary antibodies (see above), and the protein bands were detected via light emission upon HRP‐catalyzed oxidation of luminol in a LAS‐3000 (Fujifilm, Minato, Japan). Band intensities were quantified with AIDA 2D densitometry (Elysia‐Raytest, Angleur, Belgium).

### Immunofluorescence

U2OS cells were transfected as indicated in Fig. 6. Approximately 24 h after the transfection, cells were fixed in ice‐cold 100% methanol, permeabilized with 0.5% Triton X‐100, blocked with medium to reduce unspecific antibody binding, and subsequently incubated with primary and fluorochrome‐conjugated secondary antibodies (see above). An Axioplan II microscope system (Carl Zeiss, Oberkochen, Germany) with a Plan‐NEOFLUAR 100×/1.30 NA oil objective and a SPOT RT Monochrome camera (Diagnostic Instruments, Sterling Heights, MI, USA) was used for analysis and image acquisition. To determine the percentage of cells with recruitment of TNKS into AXIN1 condensates (Fig. 6B), merged images overlaying the green AXIN1 signal with the red TNKS signal were prepared, and the double positive cells on these images were characterized as either ‘cell with co‐localization’, indicative for recruitment, or ‘cell without co‐localization’. For every setup, 200 merged images from four independent experiments were prepared, resulting in about 350 analyzed cells per setup. Recruitment of AXIN2 into AXIN1 condensates (Fig. 6D) was quantified similarly.

### Statistical analysis

To probe the datasets for statistical significance of differences two‐tailed Student's *t*‐tests were performed in a non‐paired (Fig. 1A,C,D) or paired fashion (all other experiments), depending on the experimental setup. Based on graphical assessment and the assay types, normal distribution of the data was assumed, although this was not formally tested because of the small sample sizes. Values of *N* are explicitly stated where appropriate for all experiments, and statistical significance is also indicated (*P < 0.05, **P < 0.01 and ***P < 0.001), when required. *P* < 0.05 was considered statistically significant.

## Conflicts of interest

DBB was approached as potential consultant by Cytovation ASA. The other authors declare that they have no conflicts of interest.

## Author contributions

OS and MB performed the experiments. DBB analyzed the data, conceived the study and wrote the manuscript.

### Peer review

The peer review history for this article is available at https://www.webofscience.com/api/gateway/wos/peer‐review/10.1111/febs.17226.

## Data Availability

The data that supports the findings of this study are available in Figs 1, 2, 3, 4, 5, 6 and Table 1 of the published article.
